# Pull-Out Strength and Bond Behavior of Prestressing Strands in Prestressed Self-Consolidating Concrete

**DOI:** 10.3390/ma7106930

**Published:** 2014-10-10

**Authors:** Wu-Jian Long, Kamal Henri Khayat, Guillaume Lemieux, Soo-Duck Hwang, Feng Xing

**Affiliations:** 1 Guangdong Province Key Laboratory of Durability for Marine Civil Engineering, College of Civil Engineering, Shenzhen University, Shenzhen 518060, Guangdong, China; 2 Faculty of Civil, Architectural and Environmental Engineering, Missouri University of Science and Technology, Rolla, MO 65409, USA; E-Mails: khayatk@mst.edu (K.H.K.); hwangso@mst.edu (S.-D.H.); 3 Cement Association of Canada, Montreal, QC J7H 1S7, Canada; E-Mail: glemieux@cement.ca

**Keywords:** pull-out bond strength, *in-situ* compressive strength, modification factor, self-consolidating concrete, prestressed concrete, uniformity

## Abstract

With the extensive use of self-consolidating concrete (SCC) worldwide, it is important to ensure that such concrete can secure uniform *in-situ* mechanical properties that are similar to those obtained with properly consolidated concrete of conventional fluidity. Ensuring proper stability of SCC is essential to enhance the uniformity of *in-situ* mechanical properties, including bond to embedded reinforcement, which is critical for structural engineers considering the specification of SCC for prestressed applications. In this investigation, Six wall elements measuring 1540 mm × 2150 mm × 200 mm were cast using five SCC mixtures and one reference high-performance concrete (HPC) of normal consistency to evaluate the uniformity of bond strength between prestressing strands and concrete as well as the distribution of compressive strength obtained from cores along wall elements. The evaluated SCC mixtures used for casting wall elements were proportioned to achieve a slump flow consistency of 680 ± 15 mm and minimum caisson filling capacity of 80%, and visual stability index of 0.5 to 1. Given the spreads in viscosity and static stability of the SCC mixtures, the five wall elements exhibited different levels of homogeneity in *in-situ* compressive strength and pull-out bond strength. Test results also indicate that despite the high fluidity of SCC, stable concrete can lead to more homogenous *in-situ* properties than HPC of normal consistency subjected to mechanical vibration.

## 1. Introduction

With the extensive use of self-consolidating concrete (SCC) worldwide [1,2,3,4,5,6,7,8,9], it is important to ensure that such concrete can secure uniform *in-situ* mechanical properties that are similar to those obtained with properly consolidated concrete of conventional fluidity. Ensuring proper stability of SCC is essential to enhance the uniformity of *in-situ* mechanical properties, including bond to embedded reinforcement, which is critical for structural engineers considering the specification of SCC for prestressed applications [10,11]. Therefore, the stability of SCC is a key property in ensuring uniform mechanical properties and adequate performance of prestressed structural elements [12].

### 1.1. Bond Strength

There is a controversy regarding bond strength of prestressing strands embedded in SCC. The experiment conducted by Holschemacher and Klug [13] showed that bond to reinforcing bars and prestressed tendons can be influenced by the flow properties of the SCC, grading of the aggregate, and content of fines in the matrix. In general, the bond stress is improved when using SCC. Koning* et al.* [14] reported that SCC can develop higher bond strength values compared with those obtained with normal vibrated concrete. Studies carried out by Chan [15] have shown that for a given compressive strength, reinforced concrete members made with SCC can develop higher bond strength than in the case of normal concrete. This enhancement of bond strength is mainly due to the high stability of SCC that can secure a denser microstructure with the reinforcement. Stability of SCC can be enhanced by using a low w/cm, reducing the maximum size of coarse aggregate (MSA), or incorporating a viscosity-modifying admixture (VMA) [16,17].

Measurement of bond strength conducted by Gibbs* et al.* [18] showed that the bonds to reinforcing bars developed using SCC are equal to those obtained with a normal vibrated concrete. Similar results were reported by Sonebi and Castel [19,20,21,22]. Other researchers, however, have found that the bond strength of SCC to reinforcement can be lower than that of normal concrete. The German Committee for Reinforced Concrete reported that pull-out test results revealed that slightly lower bond strength can be obtained with SCC compared with normal vibrated concrete [23]. When measuring transfer length on beam elements, tests revealed that the transfer length obtained on SCC beams is similar to that observed with vibrated concrete beams. Studies carried out by José R. Martí-Vargas *et al.* [24] on SCC and traditional concrete (TC) have shown that, compared with TC, a slightly higher loss of pre-stressing force and slightly greater anchorage lengths in SCC with a low water/cement ratio; however, no differences in transfer or anchorage length were detected when high strength TC and SCC were compared.

### 1.2. Top-Bar Effect

Despite its high fluidity, SCC can develop similar modification factor (top-bar effect) as that of conventional concrete when it is proportioned with sufficient static stability [13,25,26,27]. In general, properly designed SCC can exhibit high levels of workability and stability in order to ensure uniform *in-situ* properties [16].

Decrease in top-bar effect results in reducing the difference between the bond stresses determined at the top and bottom of the element. The improvement of cohesiveness, determined from surface settlement measurements, can reduce the structural defects resulting from accumulation of bleed water, rising air bubbles, and settlement of the plastic concrete around the top reinforcement. Therefore, SCC with a high level of static stability can exhibit lower top-bar effect compared with that obtained with conventional vibrated concrete [13,16,18,19,26,28].

Khayat* et al.* [16] evaluated the uniformity of bond strength of embedded reinforcing bars along the height of experimental wall elements. The top-bar factor for reinforcing bars positioned at 1.4 m from the bottom of the experimental walls was 1.4 ± 0.2 for seven of the SCC mixtures and approximately 2.0 for the control concrete and one of the SCC mixtures. In the case of highly stable concrete made with 10 mm MSA, a lower top-bar factor of 1.0 to 1.2 was obtained. Khayat* et al.* [27] also investigated the uniformity of bond strength to prestressing strands along the height of experimental wall elements. Four SCC mixtures and two conventional flowable mixtures made with similar mix proportions were used for casting experimental wall elements. All mixtures developed 1-day compressive strength greater than 40 MPa. Uniform distribution of in-place compressive strength and adequate bond to prestressing strands were obtained. The 1- and 28-day top-bar effect ratios varied between 0.9 and 1.9. The top-bar effect was shown to be sensitive to the type of VMA in use.

Schiessl and Zilch [29] investigated the bond behavior of SCC and conventional concrete. Pull-out tests were conducted in accordance to International Union of Laboratories and Experts in Construction Materials, Systems and Structures (RILEM) recommendations using specimens measuring 300 mm in height. As expected, higher bond stress was obtained when the pull-out test is carried out for vertically-positioned bars than for horizontal bars when carried out in the opposite direction as the casting direction. In terms of bond-slip relationship, specimens cast with SCC and conventional concrete showed similar behavior. Specimens cast with SCC had uniform coarse aggregate distribution and lower top-bar effect compared with the performance of conventional concrete. Significant top-bar effect was observed in the case of conventional concrete specimens due to the settlement that can occur under the effect of vibration during the casting process.

The main objective of this investigation is to evaluate the uniformity of bond strength between prestressing strands and concrete as well as the distribution of compressive strength obtained from cores along these elements.

## 2. Experimental Program

### 2.1. Mixture Proportioning and Workability Characteristics

Based on the previous investigations on various stability levels of SCC mixtures [30], six wall elements measuring 1540 mm × 2150 mm × 200 mm were selected and cast using five SCC mixtures and one reference high-performance concrete (HPC) of normal consistency to evaluate the homogeneity of bond strength between SCC and horizontally embedded prestressing strands positioned at various heights in wall elements, as shown in Table 1.

**Table 1:** Selection of self-consolidating concrete (SCC) and high-performance concrete (HPC) mixtures for strand bond test.

Type	Wall No.	Mix Proportions	Descriptions
SCC	1	34-440-HE20%FA-S/A54-VMA	Highly viscous to simulate lack of consolidation
			Plastic viscosity = 300 Pa.s
			Maximum settlement = 0.44%
	2	40-500-MS-S/A46-VMA *	Low viscosity,
			Plastic viscosity = 20 Pa.s
			Maximum settlement = 0.59%
	3	40-440-MS-S/A54-VMA	Unstable mixture,
			Plastic viscosity = 70 Pa.s
			Maximum settlement = 0.62%
	4	34-500-HE20%FA-S/A46-VMA **	Stable mixture,
			Plastic viscosity = 80 Pa.s
			Maximum settlement = 0.43%
	5	34-440-HE20%FA-S/A46	Stable mixture,
			Plastic viscosity = 150 Pa.s
			Maximum settlement = 0.3%
HPC	6	34-MS	Stable mixture,
			Plastic viscosity = 110 Pa.s
			Maximum settlement = 0.29%

* 40-500-MS-S/A46-VMA = (0.40 w/cm, 500 kg/m^3^ of binder, Type MS (moderate sulfate) cement, Sand-to-total Aggregate ratio = 0.46, incorporating of viscosity-modifying admixture (VMA)); ** 34-500-HE20%FA-S/A46-VMA = (0.34 w/cm, 500 kg/m^3^ of binder, Type HE (high early strength) cement + 20% fly ash, Sand-to-total Aggregate ratio = 0.46, incorporating of VMA).

The mixture proportioning and workability characteristics of the mixtures used to cast of wall elements are summarized in Table 2. Despite different stability levels of the evaluated mixtures, five SCC mixtures had similar level of slump flow consistency of 660 to 695 mm and adequate caisson filling capacity of 82% to 93%. The surface settlement of the SCC mixtures ranged between 0.30% and 0.62% with a target value of 0.50%. The maximum surface settlement of the HPC mixture was 0.29%.

In addition to the pull-out strength test, three core samples measuring 95 mm in diameter and 200 mm in height were taken at each height corresponding to that of the embedded strand. In-place compressive strength results determined from core samples were compared with those of reference cylinders cured in the same manner as wall element.

**Table 2:** Mixture proportioning and workability characteristics of tested concretes.

Wall No.	SCC 1	SCC 2	SCC 3	SCC 4	SCC 5	HPC 6
Cement, kg/m^3^	Type HE	Type MS	Type MS	Type HE	Type HE	Type MS
	352	500	440	400	352	470
HRWRA * demand, L/100 kg CM **	3.35	0.60	1.00	2.00	3.00	0.50
VMA dosage, L/100 kg CM	0.1	0.1	0.1	0.1	0	0
Class F fly ash, kg/m^3^	88	0	0	100	88	0
Water, kg/m^3^	139	196	172	161	139	160
w/cm	0.34	0.40	0.40	0.34	0.34	0.34
Sand, kg/m^3^	984	762	955	789	839	741
Coarse aggregate, kg/m^3^	838	894	814	927	985	1050
Sand/total aggregate, by volume	0.54	0.46	0.54	0.46	0.46	0.41
Slump flow, mm	665	695	670	660	660	145 ***
T-50 mm, s	6.8	1.5	1.8	2.9	5.5	–
Visual stability index	0.5	1	1	0.5	0.5	0
Air content, %	2.4	2.3	1.9	2.0	1.1	2.7
Unit weight, kg/m³	2343	2352	2365	2360	2409	2429
Temperature, °C	23	24	23	24.4	23.5	25
J-Ring, mm	630	640	605	630	605	–
Filling capacity, %	92	93	89	91	82	–
Maximum surface settlement, %	0.44	0.59	0.62	0.43	0.30	0.29
Yield stress, Pa	5	32	37	25	12	575
Plastic viscosity, Pa.s	300	20	70	80	150	110

* HRWRA = high-range water-reducing admixture; ** CM = cementitious materials; *** Slump.

### 2.2. Bond Strength Measurement of Prestressing Strands

**Figure 1:**
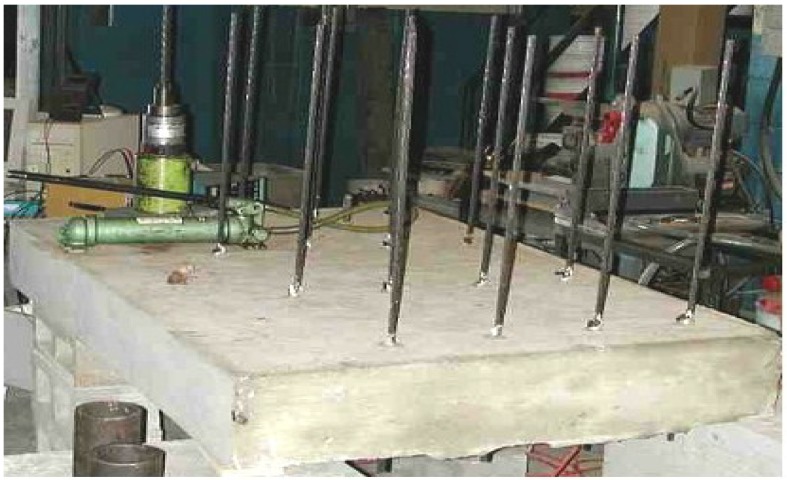
Prestressing strands embedded at various heights of wall element.

**Figure 2:**
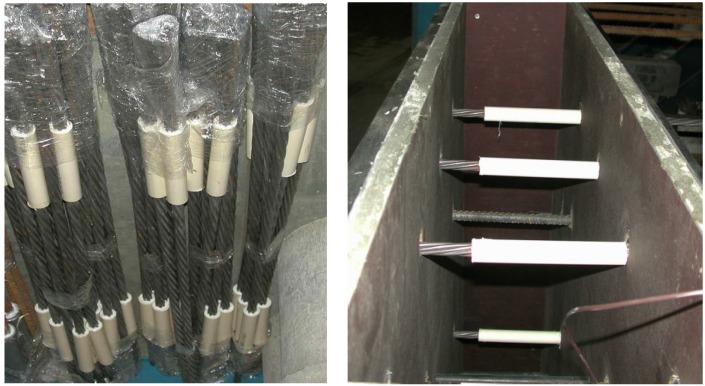
Polyvinyl chloride polymer (PVC) sheathing and installation of prestressing strands.

An experimental wall measuring 1540 mm × 2150 mm × 200 mm was used for the bond strength testing. The formwork was divided in two parts, each cast with different concrete. Testing consisted of determining the maximum pull-out load* vs.* the end slip response of strands that are horizontally embedded in experimental wall elements. As presented in Figure 1, each wall had 16 Grade 270 low-relaxation prestressing strands of 15.2 mm diameter grouped in four strands per row. The strands were positioned at four different heights. Rigid plastic sheathing was attached to the outer end of each strand near the loaded end as bond breaker to minimize secondary confining stresses along the bonded region, as shown in Figure 2.

**Figure 3:**
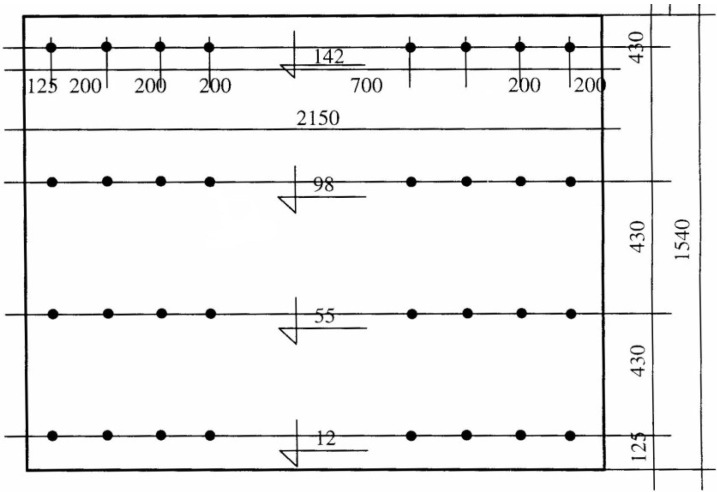
Detail dimensions of experimental wall element (mm).

The clear cover over exterior strands was 125 mm, and the center-to-center spacing between adjacent strands was 200 mm. The details of the wall section and the position of the prestressing strands are given in Figure 3.

**Figure 4:**
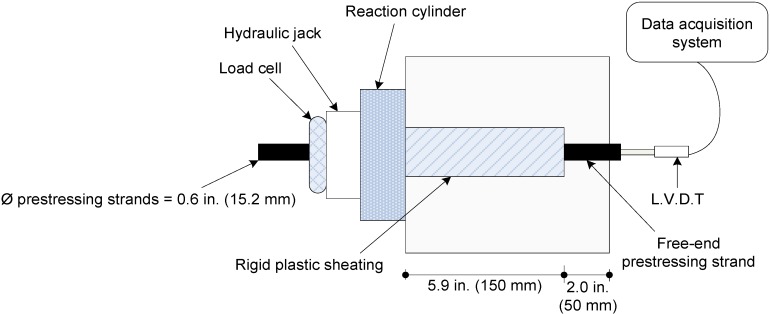
Schematic of pull-out testing arrangement.

**Figure 5:**
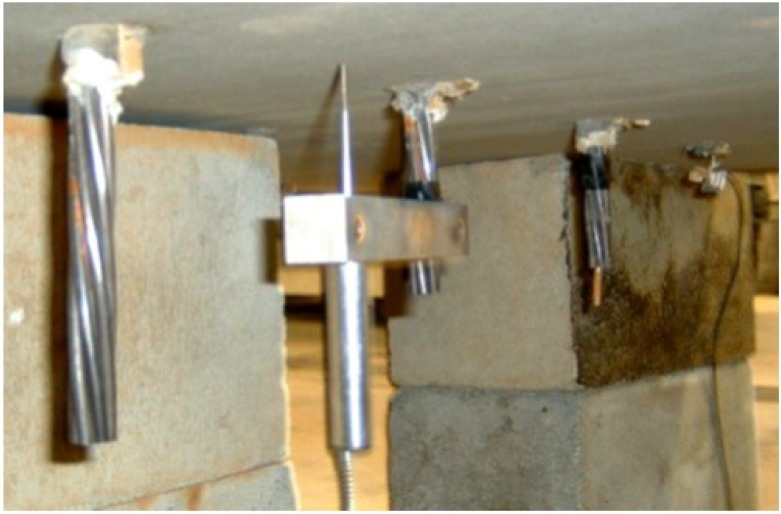
Monitoring of free-end slip of prestressing strand.

The wall elements were removed from the mold at the age of one day, moist-cured for 7 days, and kept under ambient temperature conditions until the age of 56 days. Pull-out tests were carried out at 56 days after casting. The wall elements were gently tilted onto an elevated and horizontal platform to facilitate testing. A hydraulic jack with 135-kN capacity was used for loading. As presented in Figure 4, the hydraulic jack was attached to the prestressing strands, and a reaction cylinder was positioned against the concrete. Pull-out load was applied gradually. The net slip of the prestressing strand was monitored using an Linear Variable Differential Transformer (LVDT) attached to the unloaded end of the strand (Figure 4 and Figure 5). After debonding, the prestressing strands were completely extracted to determine exact anchorage length necessary for calculation of bond stress.

**Figure 6:**
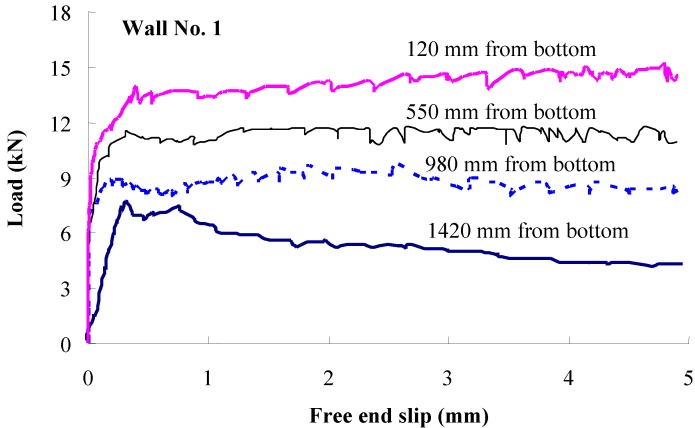
Pull-out load and free-end slip responses of prestressing strands embedded at different heights along wall element (Wall No. 1).

Variations of pull-out load and free-end slip results for Wall No. 1 cast with SCC No. 4 are presented in Figure 6. Mean values of pull-out load and free-end slip from four prestressing strands at each height (120, 550, 980, and 1420 mm from the bottom) were plotted in Figure 6.

## 3. Results and Discussion

### 3.1. In-Place Compressive Strength

**Figure 7:**
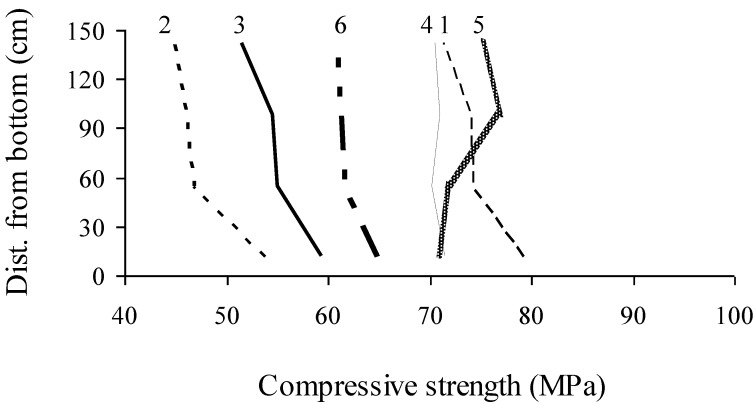
Distribution of in-place compressive strength at 56 days determined from core samples along experimental wall elements.

In addition to the pull-out test, three core samples measuring 95 mm in diameter and 200 mm in height were taken at each height corresponding to that of the embedded strand. In-place compressive strength results determined from core samples were compared with those of reference cylinders cured under similar conditions of the wall element. In-place compressive strength values of core samples taken at various heights along the wall are presented in Figure 7 and Table 3. Walls No. 1, 4, and 5 exhibited higher in-place compressive strength values compared with Walls No. 2, 3, and 6. Wall No. 4 showed uniform distribution of compressive strength along the height of the wall.

**Table 3:** In-place compressive strength of wall elements cast with SCC and HPC with different stability levels.

Mix. No	Distance from Bottom, mm	Compressive Strength, MPa					f'c core/f'c cylinder (%)
		1	2	3	Mean	C.O.V. * (%)	
Wall No. 1	120	81.9	77.9	78.3	79.4	2.79	98
	550	71.0	77.1	75.1	74.4	4.16	92
	980	73.8	74.4	74.4	74.2	0.49	92
	1420	75.1	63.1	76.5	71.5	10.27	88
	Cylinder	81.3	80.7	80.6	80.9	0.48	100
Wall No. 2	120	55.9	55.0	50.7	53.9	5.21	99
	550	48.1	45.4	47.3	46.9	2.87	86
	980	44.6	45.9	47.8	46.1	3.40	84
	1420	43.3	45.9	45.5	44.9	3.23	82
	Cylinder	54.1	53.8	55.7	54.6	1.86	100
Wall No. 3	120	60.4	59.0	58.8	59.4	1.44	98
	550	55.7	54.4	55.1	55.1	1.19	91
	980	52.9	54.0	56.6	54.5	3.54	90
	1420	51.4	51.4	51.7	51.5	0.25	85
	Cylinder	60.8	59.7	60.5	60.3	0.90	100
Wall No. 4	120	71.0	70.0	73.8	71.6	2.76	95
	550	70.9	68.9	71.0	70.3	1.71	93
	980	74.4	66.4	72.6	71.1	5.90	94
	1420	72.2	69.8	69.7	70.6	1.97	94
	Cylinder	76.8	78.3	71.3	75.5	4.86	100
Wall No. 5	120	75.2	69.6	68.1	71.0	5.34	91
	550	74.2	72.1	69.1	71.8	3.60	92
	980	77.8	80.9	72.1	77.0	5.75	99
	1420	74.3	74.8	76.2	75.1	1.33	96
	Cylinder	78.9	78.0	76.8	77.9	1.37	100
Wall No. 6	120	64.8	63.9	66.0	64.9	1.66	99
	550	64.7	61.7	59.0	61.8	4.59	94
	980	62.0	61.2	61.2	61.5	0.70	94
	1420	62.0	58.3	62.5	60.9	3.79	93
	Cylinder	64.4	64.5	68.3	65.7	3.36	100

* C.O.V. = coefficient of variation.

As presented in Figure 7, walls No. 1, 4, and 5 developed higher in-place compressive strength than Wall No. 6. This is mainly due to the difference in binder type used (type HE and 20% of fly ash comparatively to type MS).

**Figure 8:**
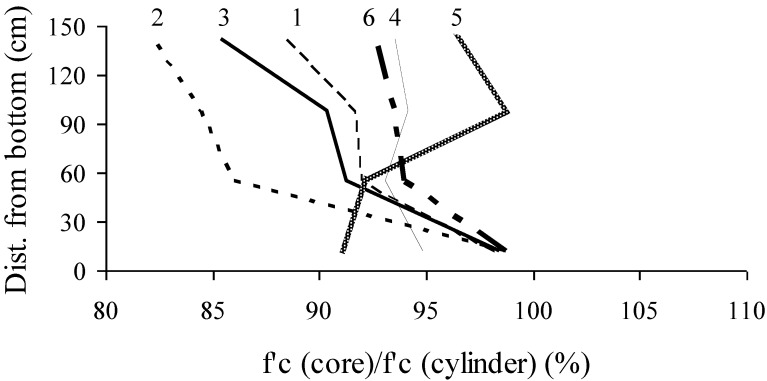
Variations in relative in-place compressive strengths with height (strength ratio relative to reference cylinder).

In-place compressive strength values of core samples taken from various heights are compared with those determined from companion cylinders (Figure 8), which were kept in their molds for one day before being demolded. These cylinders were then stored in an environment similar to the wall sections and were tested at the same age as cores. As expected, Walls No. 4, 5, and 6 cast with stable mixtures exhibited more homogenous in-place compressive strength compared with Wall No. 1, 2, and 3. On average, relative in-place compressive strength was about 90% of values obtained with the control cylinders. This is typical for the core samples tested in compression in a perpendicular direction to the casting position. Walls No. 1 to 3 had in-place strength ratios lower than 90% at the top of the walls given their lower level of stability that hinders uniform distribution of *in-situ* properties of hardened concrete.

**Figure 9:**
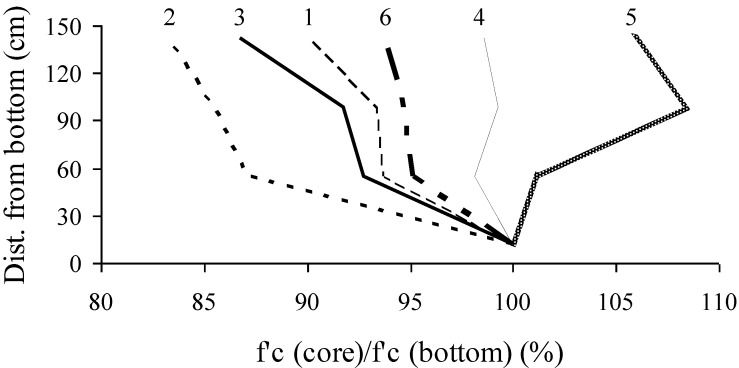
Variations in in-place compressive strengths with height (strength ratio relative to bottom layer).

Core strength values normalized to those of core samples taken near the bottom of the wall elements are plotted in Figure 9. Wall elements 1, 2, and 3 cast with unstable mixtures had in-place compressive strength of 90% ± 5% relative to strength values of core samples taken near the bottom. On the other hand, these values were 100% ± 6% for Wall elements 4, 5, and 6 made with stable SCC and HPC with conventional slump.

### 3.2. Pull-Out Bond Strength and Modification Factor (Top-Bar Factor)

**Figure 10:**
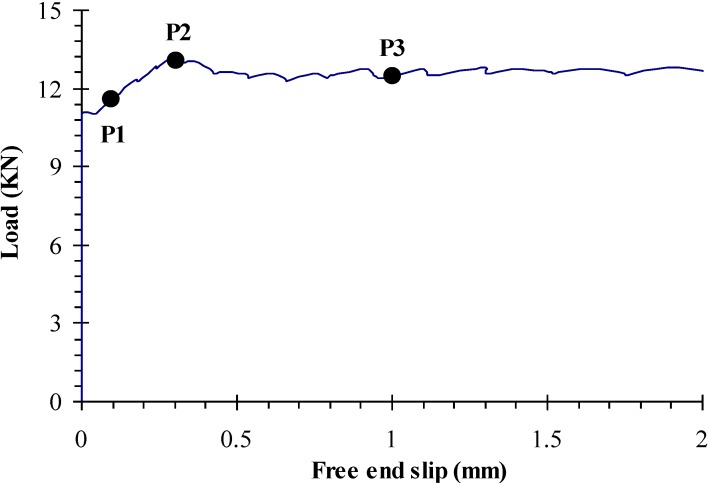
Definition of pullout loads corresponding to bond strength comparisons.

Bond strength results of prestressing strands determined at 56 days of age are summarized in Table 4. Three load levels are considered, as illustrated in Figure 10. The P1 load reflects the force corresponding to de-cohesion and can be used to evaluate bond strength at the end of elastic region. This de-cohesion is taken at a given deformation of 0.1 mm. After P1, there is an increase in load carrying capacity and cracking surrounding concrete. The P2 load corresponds to the maximum load at the free-end deformation less than 1 mm. Depending on the case, this load can be slightly higher than 1 mm deformation (P3). The P3 load at free-end slip of 1 mm is taken to calculate bond strength in the post elastic cracked region. In general, the free-end slip corresponding to the P2 load was smaller than that of the P3 load. In some mixtures, however, it was hard to select or find the peak point corresponding to the P2 load due to the continuous increase in pull-out load. Therefore, the P3 load corresponding to the free-end slip of 1mm was used to calculate bond strength in the post elastic cracked region.

**Table 4:** Bond strength and top-bar effect of prestressing strands at 56 days of age.

Mix. No	Distance From Bottom, mm	Average Bond Strength, MPa	Normalized Bond Strength, MPa^1/2^	Normalized Top-Bar Effect
		P3 level	U_P3_	P3 level
Wall No. 1	120	5.5	0.62	1.00
	550	3.3	0.37	1.61
	980	3.1	0.36	1.75
	1420	2.8	0.33	1.85
Wall No. 2	120	4.7	0.64	1.00
	550	4.1	0.60	1.05
	980	2.7	0.40	1.57
	1420	2.7	0.40	1.57
Wall No. 3	120	5.7	0.74	1.00	
	550	5.4	0.73	1.02	
	980	4.0	0.54	1.37	
	1420	2.8	0.39	1.88	
Wall No. 4	120	5.3	0.63	1.00	
	550	5.5	0.66	0.96	
	980	5.6	0.66	0.95	
	1420	5.3	0.63	1.00	
Wall No. 5	120	7.3	0.87	1.00	
	550	7.4	0.87	1.00	
	980	7.0	0.80	1.10	
	1420	7.3	0.84	1.03	
Wall No. 6	120	8.7	1.08	1.00	
	550	8.3	1.06	1.02	
	980	7.1	0.91	1.19	
	1420	6.2	0.79	1.36	

**Figure 11:**
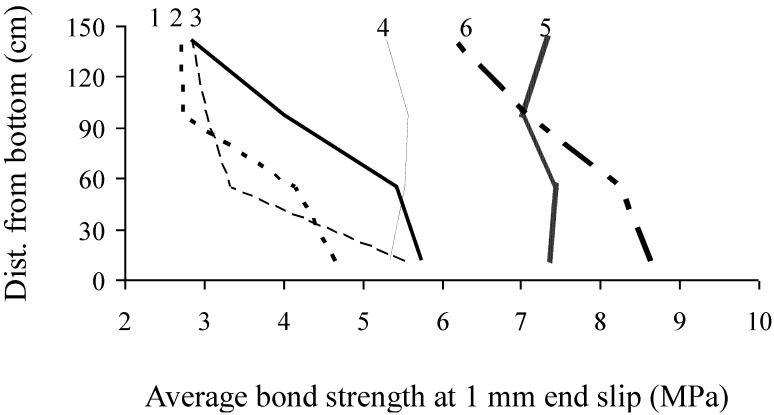
Variation in bond strength of prestressing strands along wall height.

Variations of bond strength at 1 mm end slip are presented in Figure 11. Walls No. 4 and 5 cast with stable SCC mixtures exhibited more homogenous pull-out bond strengths along the height compared with walls cast with unstable SCC. Walls No. 4 and 5 exhibited even better homogeneity in pull-out strength than Wall No. 6 cast with HPC mixture. It is important to note that Wall No. 1 exhibited relatively large spread in pull-out bond strength along the height. This can be attributed to the significantly high plastic viscosity of 300 Pa.s, which seems to hinder self-consolidation. Among the six tested walls, Wall No. 4 had more homogenous *in-situ* bond strength than the other walls. SCC wall No. 4 had adequate level of plastic viscosity of 80 Pa.s and maximum settlement of 0.43%.

**Figure 12:**
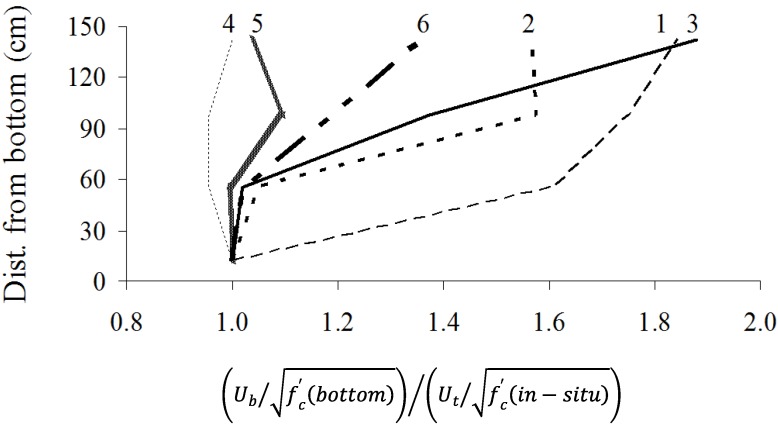
Variation of modification factor of prestressing strands along wall height.

Variations of the modification factors of bond strength between the concrete and prestressing strands are illustrated in Figure 12. Bond strength values are normalized by the square root of compressive strength determined from core samples at the corresponding strand heights. Normalized bond strengths are compared with those from the bottom layer. From the previous experience on the pull-out bond strength, top-bar effect can vary between 0.9 and 1.9 for the concrete of normal consistency, and between 1.3 and 2.0 for the SCC [16,25].

In general, Walls No. 4, 5, and 6 cast with stable SCC and HPC exhibited lower modification factor of 1, 1, and 1.36, respectively, compared with 1.57 and 1.88 for Walls No. 2 and 3 cast with unstable mixtures, respectively. Wall No. 1 exhibited a relatively large spread in modification factor values along the height. Again, this can be attributed to the lack of consolidation leading to an undesirable bond between concrete and prestressing strand. It is interesting to note that Walls No. 4 and 5 exhibited lower modification factors than Wall No. 6 made with HPC mixture. Wall 6 had a higher modification factor of 1.36 compared with 1.0 to 1.1 along height for Walls 4 and 5 cast with stable SCC mixtures. This reflects the highly stable nature of these SCC mixtures of moderate viscosity levels that enabled full adequate self-consolidation and reduction in surface settlement.

### 3.3. Effect of Stability on Homogeneity of In-Place Compressive and Bond Strength

**Figure 13:**
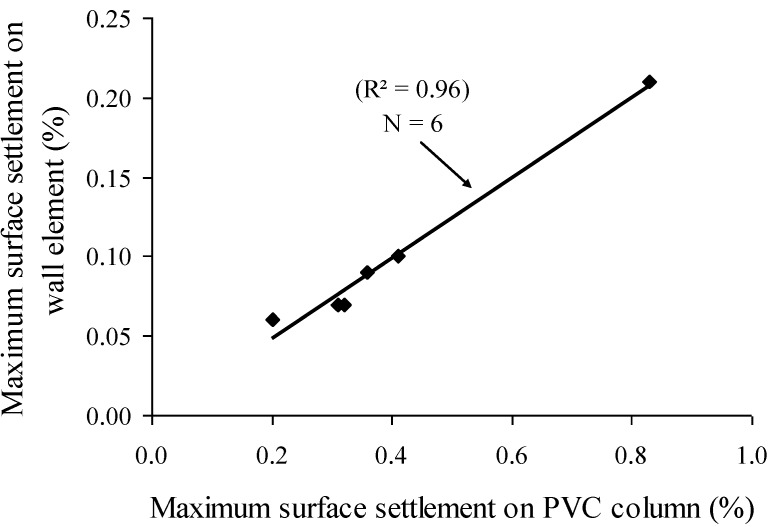
Relationship between surface settlement results determined on PVC columns and wall elements.

As a static stability index, surface settlement of concrete was determined using PVC columns filled with concrete of 660 mm as well as at the top of the wall element measuring 1540 mm × 2150 mm × 200 mm. Test results showed that, the maximum surface settlement determined on the wall element was approximately four times lower than those obtained on the PVC column testing (Figure 13). The two settlement values determined from different methods exhibited very high correlation factor (*R*^2^ = 0.96), as presented in Figure 13. Thus, the determination of surface settlement using the PVC test device can then be used to estimate the relative static stability of the various mixtures cast in the wall elements.

**Figure 14:**
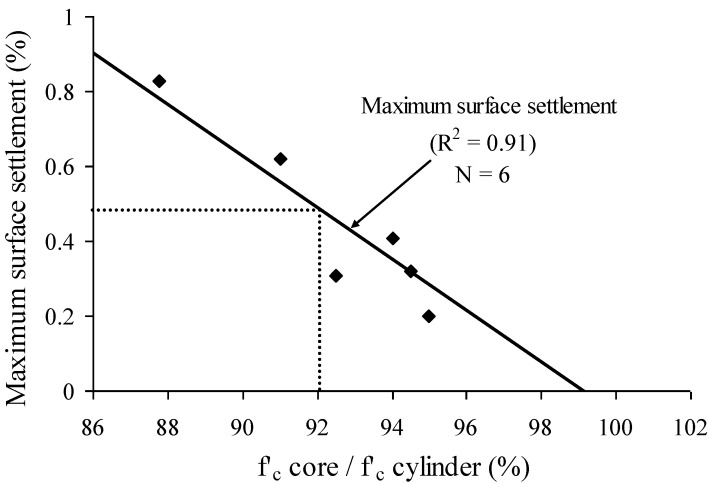
Relationship between relative in-place compressive strength (strength ratio relative to reference cylinder) and maximum surface settlement determined on PVC column.

Surface settlement of concrete was shown to have a considerable influence on the in-place compressive strength ratio relative to reference cylinders. As presented in Figure 14, the relative in-place compressive strength increased with the decrease in maximum surface settlement with a high *R*^2^ value of 0.91. Given the relationship in Figure 14, concrete having a maximum surface settlement lower than 0.5% can develop relative in-place compressive strength (core/cylinder) higher than 0.92.

## 4. Conclusions

Based on the test results and discussion above, the following conclusions can be drawn:

- Wall elements cast with stable mixtures exhibited more homogenous in-place compressive strengths and pull-out bond strengths compared with walls cast with unstable mixtures. On average, relative in-place compressive strength was about 90% of values obtained with the control cylinders.
- Walls cast with stable SCC and HPC exhibited lower modification factor between 1 and 1.36, whilst those cast with unstable mixtures exhibited a modification factor between 1.57 and 1.88. Despite the high fluidity of SCC, stable concrete can lead to more homogenous *in-situ* properties than HPC of normal consistency subjected to mechanical vibration.
- The recommendations to ensure homogenous *in-situ* properties are as follows: the concrete should have maximum surface settlement lower than 0.5%; plastic viscosity up to 160 Pa.s; relative compressive strength ratio (core to cylinder) higher than 90%; and top-bar effect lower or equal to 1.4. It is important to note that the selection of more viscous mixtures needs extra care to ensure an acceptable consolidation of the concrete. The lack of consolidation can lead to low bond stress between concrete and prestressing strand.
